# Association between complete blood count-derived hematological inflammatory ratios and nutritional risk in elderly patients with acute ischemic stroke

**DOI:** 10.3389/fneur.2026.1821874

**Published:** 2026-09-18

**Authors:** Liang’e Xu, Wenjing Wu, Chao Chen

**Affiliations:** 1 Department of Neurology, The Third Affiliated Hospital of Wenzhou Medical University, Wenzhou, China; 2 Department of Neurology, The Second Affiliated Hospital of Zhejiang Chinese Medical University, Hangzhou, China; 3 Department of Clinical Nutrition, The Third Affiliated Hospital of Wenzhou Medical University, Wenzhou, China

**Keywords:** acute ischemic stroke, CBC-derived hematological inflammatory ratios, elderly patients, monocyte-to-lymphocyte ratio, nutritional risk

## Abstract

**Background and aims:**

Mounting evidence suggests CBC-derived hematological inflammatory ratios correlate with nutritional status, yet few studies explore their cross-sectional associations with GNRI-defined concurrent nutritional risk in elderly patients with acute ischemic stroke (AIS). This single-center, cross-sectional, observational study was designed to evaluate the correlation between routine admission hematological markers and nutritional risk in elderly patients with AIS. Twelve CBC-derived indices were analyzed: lymphocyte ratio (LR), red blood cell-to-lymphocyte ratio (RLR), hemoglobin-to-lymphocyte ratio (HLR), monocyte-to-lymphocyte ratio (MLR), monocyte-to-neutrophil ratio (MNR), neutrophil-to-lymphocyte ratio (NLR), NLR-to-platelet ratio (NLR/PLT100), MLR-to-platelet ratio (MLR/PLT), platelet-to-neutrophil ratio (PNR), platelet-to-lymphocyte ratio (PLR), mean platelet volume-to-lymphocyte ratio (MPVLR), serum albumin-to-lymphocyte ratio (ALBLR).

**Methods:**

Between January 2022 and January 2024, 540 elderly AIS patients (≥60 years) were enrolled. All participants underwent admission GNRI assessment and were split into GNRI-defined nutritional risk group (GNRI≤98, *n* = 245, 45.4%) and non-nutritional risk group (GNRI>98, *n* = 295). Venous blood was collected within 24 h on admission. Univariate and multivariate logistic regression were applied to explore cross-sectional correlations, and an exploratory combined statistical model was built. ROC curve, DeLong’s test, likelihood ratio test and AIC were used to evaluate model discrimination and fit.

**Results:**

Multiple indicators including age, hypertension, ALT, UA, TG, LR, RLR, MLR, NLR, NLR/PLT100, MLR/PLT, PLR and MPVLR showed univariate correlations with nutritional risk. Multivariate regression identified MLR and NLR/PLT100 as independent correlates within this cohort; MLR showed the strongest association in this dataset instead of robust correlative ability. Adding MLR alone or combining MLR + NLR/PLT100 to base clinical variables significantly improved C-statistic. The combined model reached an AUC of 0.735, representing only moderate discriminative capacity, and it was merely the superior exploratory model limited to the present sample without external generalizability.

**Conclusion:**

Within this single-center cohort, MLR exhibited the strongest cross-sectional correlation with GNRI-defined nutritional risk among all tested CBC-derived hematological inflammatory ratios. The composite model combining baseline clinical indicators, MLR and NLR/PLT*100 demonstrated moderate discriminative ability within the study sample, but is not yet robust enough to serve as an effective tool for routine clinical risk stratification.

## 1 Introduction

Stroke poses a significant global health burden, ranking as the second leading cause of mortality and the third leading cause of disability in adults worldwide. In China, ischemic stroke is the predominant subtype, accounting for approximately 70% of all stroke cases (1, 2). Ischemic stroke can be classified into different stages based on the time of onset, with the acute phase typically referring to the first 2 weeks after the onset, and in clinical research, it is often defined as within 48 h of the onset. Acute ischemic stroke (AIS) patients frequently develop dysphagia, neurological deficits, cognitive impairment, and sensorimotor or visual field disturbances, all of which impair oral intake or completely prevent adequate food consumption. Combined with stroke-induced hypermetabolism, these impairments greatly increase the likelihood of nutritional risk, a pre-clinical state of insufficient nutrient reserve that precedes overt malnutrition. We provide clear differentiation among three frequently conflated nutritional terms to standardize subsequent narrative: nutritional risk represents a screening-identified at-risk state captured by GNRI, which only signals elevated probability of subsequent malnutrition and does not equate to a definitive clinical diagnosis of malnutrition; poor nutritional status acts as an umbrella term covering both nutritional risk and confirmed malnutrition; malnutrition refers to formal clinical nutrient depletion diagnosed via standardized frameworks such as GLIM criteria, reflecting persistent and severe nutritional deficiency. Post-stroke malnutrition significantly increases the risk of stroke-related complications, including pneumonia, urinary tract infections, deep vein thrombosis (DVT), and gastrointestinal bleeding, prolongs hospitalization, and ultimately compromises recovery and long-term functional prognosis (3). Multiple feeding and nutritional trials confirm that malnutrition is an independent risk factor for 6-month mortality and unfavorable functional outcomes in AIS patients (4).

Recently, attention has focused on readily available inflammatory hematological markers, including the neutrophil-to-lymphocyte ratio (NLR), platelet-to-lymphocyte ratio (PLR), mean platelet volume-to-lymphocyte ratio (MPVLR), monocyte-to-lymphocyte ratio (MLR), and mean platelet volume-to-platelet ratio (MPR). These biomarkers are characterized by their cost-effectiveness, accessibility, and non-invasiveness. NLR and PLR, which reflect the equilibrium of immune responses, have been identified as independent and cost-efficient correlates for various inflammatory and immune-mediated conditions, such as pneumonia, coronary heart disease (CHD), and malignancies (5, 6). MLR has been established as a valuable predictor for systemic inflammation and infections—including community-acquired pneumonia, osteoporosis, acquired immune deficiency syndrome (AIDS), malaria, and coronary artery disease (CAD) severity—while also serving as a prognostic factor for solid tumors (7). Emerging evidence suggests that these lymphocyte-related ratios are not only markers of systemic inflammation but also potent correlates of nutritional risk. For instance, recent studies have demonstrated a significant correlation between elevated NLR or Systemic Immune-Inflammation Index (SII) and poor nutritional status as assessed by the Controlling Nutritional Status (CONUT) score or Prognostic Nutritional Index (PNI) in patients with ischemic stroke (8). Furthermore, combined indices such as the HALP score (hemoglobin, albumin, lymphocyte, and platelet) have shown promising prognostic value in predicting outcomes for critically ill elderly patients, further underscoring the interplay between inflammation, immunity, and nutrition (9). Although previous studies have confirmed that NLR can predict early neurological deterioration in stroke patients and serve as a screening tool for nutritional risk in elderly individuals, t the association between a full panel of CBC-derived hematological inflammatory ratios and nutritional risk in elderly stroke patients remains unexplored (10, 11).

We elaborate the biological rationale guiding the selection of the 12 hematological ratios assessed in this study while addressing potential circularity concerns raised by albumin- and hemoglobin-containing indices. Existing relevant studies only reported single or 2–3 inflammatory ratios related to stroke nutritional status, without systematic head-to-head comparison of a complete CBC-derived ratio panel targeting elderly AIS populations. To fill this literature gap and conduct comprehensive exploratory screening for optimal correlated markers, we incorporated all 12 indices rather than selecting a small pre-defined panel based on limited prior evidence. These 12 indicators were divided into four functionally distinct categories: (1) Core inflammation-lymphocyte balanced ratios (LR, MLR, NLR, PLR, MPVLR, RLR): mirror the equilibrium between inflammatory myeloid cells, platelets, red blood cells and lymphocytes to reflect post-stroke inflammaging and immune suppression, two core pathways linking inflammatory activation to progressive nutrient depletion; (2) Novel platelet-adjusted composite indices NLR/PLT*100 and MLR/PLT: eliminate confounding effects from abnormal platelet counts, representing rarely explored markers with exploratory value in stroke nutritional research; (3) Nutrient molecule-lymphocyte composite indices HLR and ALBLR: test whether combining core nutritional molecules hemoglobin and albumin with lymphocyte counts delivers incremental nutritional information beyond standalone albumin or hemoglobin measurements; (4) Myeloid-platelet indices MNR and PNR: lack lymphocyte denominators and serve as auxiliary contrast markers to complete the peripheral immune profiling of patients facing nutritional risk. Statistical multicollinearity diagnostic strategies (VIF filtering, hierarchical regression, forward stepwise variable screening) were strictly implemented in subsequent analyses to offset inherent mathematical correlation among overlapping ratios and guarantee reliable identification of independent correlates. Classic inflammation-lymphocyte balanced ratios including LR, MLR, NLR, PLR, MPVLR and RLR form the core panel of indicators, as they mirror the equilibrium between inflammatory myeloid cells, platelets, red blood cells and lymphocytes to reflect post-stroke inflammaging and immune suppression, two core pathways linking inflammatory activation to progressive nutrient depletion. Novel platelet-adjusted composite indices NLR/PLT*100 and MLR/PLT are incorporated to eliminate confounding effects from abnormal platelet counts, representing rarely explored markers with exploratory value in stroke nutritional research. HLR and ALBLR are included to test whether combining core nutritional molecules hemoglobin and albumin with lymphocyte counts delivers incremental nutritional information beyond standalone albumin or hemoglobin measurements. We acknowledge the risk of analytical circularity associated with ALBLR given albumin is a core component of GNRI, though partial mitigation exists as hemoglobin is not incorporated into GNRI calculation; to avoid biased interpretation, we adopt stratified regression models in subsequent analyses to separate the overlapping nutritional signal of albumin shared by ALBLR and GNRI. Supplementary myeloid-platelet indices MNR and PNR are added to complete the peripheral immune profiling of patients facing nutritional risk.

We further elaborate the distinct practical clinical advantages of CBC-derived hematological inflammatory ratios compared with sole GNRI screening to clarify the clinical application value of these complete blood count (CBC)-derived markers. Standard GNRI calculation requires manual collection of height, weight and serum albumin, yet anthropometric data are often unavailable or delayed in emergency AIS admissions complicated by severe aphasia, critical unstable condition or inability to complete body weight measurement. By contrast, CBC testing is routinely ordered for all acute stroke patients upon emergency admission, and all 12 CBC-derived hematological inflammatory ratios can be automatically calculated from routine CBC results without additional blood sampling, extra laboratory expenditure or manual anthropometric measurement. In urgent clinical scenarios where height and weight cannot be immediately obtained, CBC-derived hematological inflammatory ratios serve as rapid auxiliary screening surrogates to quickly identify patients with high nutritional risk prior to formal GNRI assessment. Even when complete anthropometric and albumin data are accessible for GNRI calculation, these hematological ratios provide unique inflammatory and immune information that cannot be captured by GNRI alone, supporting multidimensional risk stratification integrating both nutritional reserve and systemic inflammatory burden, which delivers more comprehensive risk evaluation for elderly AIS patients complicated by robust post-stroke inflammatory response.

### 1.1 Clinical advantages of CBC-derived inflammatory markers vs. GNRI screening

The routine GNRI nutritional assessment relies on height, weight and serum albumin, which are frequently unmeasurable during emergency admission for critical aphasic or unstable elderly AIS patients. All 12 lymphocyte-related hematological ratios can be automatically calculated from routine admission CBC without extra blood drawing, additional lab costs or manual body measurement, enabling rapid preliminary nutritional risk triage when full GNRI data are temporarily unavailable. Moreover, CBC inflammatory ratios reflect systemic immune-inflammation status that GNRI cannot capture, supporting multi-dimensional risk evaluation combining nutrition and inflammation.

### 1.2 Explicit study hypotheses

Primary hypothesis: MLR shows the strongest correlation with GNRI nutritional risk, as it jointly reflects post-stroke inflammation and malnutrition-related lymphopenia. Secondary hypothesis: Combining MLR + NLR/PLT*100 can significantly improve model discrimination compared with the basic clinical model.

### 1.3 Independent clear study objectives

Therefore, this single-center cross-sectional observational study aimed to systematically investigate associations between the 12 CBC-derived hematological inflammatory ratios and GNRI-defined nutritional risk rather than formal malnutrition diagnosis in elderly AIS patients, identify candidate independent correlates, and construct an exploratory combined screening model to facilitate rapid clinical risk stratification.

## 2 Materials and methods

### 2.1 Study population

This was a single-center, retrospective observational study with consecutive chart review of elderly patients admitted for first-ever AIS between January 2022 and January 2024. A total of 1,097 cases were initially screened. Based on the onset time, age, first stroke, imaging diagnosis criteria, and exclusion criteria (such as concurrent tumors, severe heart, liver, and kidney dysfunction, immune inflammatory diseases, active infections, etc.), a step-by-step screening was conducted. Eventually, 540 elderly patients (≥60 years old) who met the criteria were included. The inclusion criteria were as follows: (1) First-ever AIS patients admitted within 48 h of onset, with diagnosis established according to the Chinese Guidelines for Diagnosis and Treatment of AIS and confirmed by neuroimaging (CT or MRI) (12); (2) age ≥60 years; and (3) provision of written informed consent from either the patients or their legal guardians. Patients were excluded if they presented with any of the following conditions: (1) history of other neurological disorders within 3 months prior to AIS onset; (2) concurrent malignancy; (3) recent acute myocardial infarction; (4) rheumatic immune diseases; (5) severe hepatic or renal dysfunction; (6) acute or chronic immune-inflammatory dis-eases; or (7) active infectious diseases. The detailed selection criteria of the study sub-jects were demonstrated in Figure 1.

**Figure 1:**
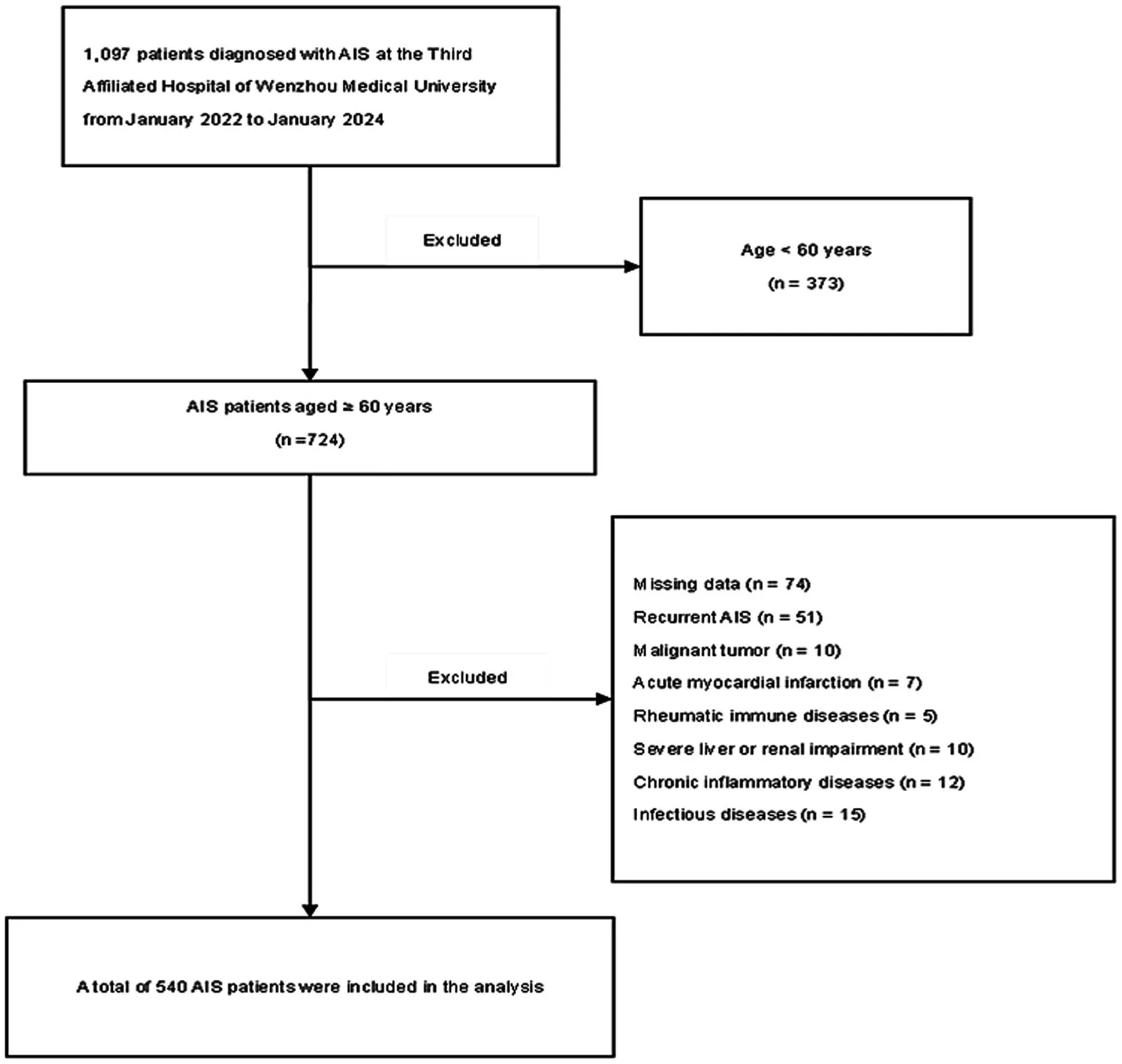
Flowchart of the study population selection.

### 2.2 Data collection

Baseline demographic and clinical characteristics, including gender, age, height, weight, systolic blood pressure (SBP), diastolic blood pressure (DBP), and history of hypertension, were extracted from the electronic medical records (EMR) (13). All venous blood samples for CBC and serum biochemistry were collected prior to any emergency intervention, including intravenous fluid resuscitation, parenteral nutritional support, antibiotic administration, intravenous thrombolysis, endovascular thrombectomy, and corticosteroid therapy, within 24 h of emergency admission to eliminate interference of post-intervention inflammatory and metabolic fluctuations on hematological ratios. CBC parameters were analyzed using the XT-1800i analyzer (Sysmex, Kobe, Japan), while blood biochemical indices were measured using the ARCHITECT c16000 system (Abbott Laboratories, Illinois, United States). All laboratory assays were performed within 24 h of admission.

The Geriatric Nutritional Risk Index (GNRI) was adopted to screen GNRI-defined nutritional risk rather than confirm formal clinical malnutrition diagnosis. GNRI is an objective geriatric nutritional screening tool built upon serum albumin concentration and anthropometric weight parameters, and it cannot serve as a comprehensive diagnostic standard for malnutrition per GLIM criteria. The GNRI was computed as follows: GNRI = (1.489 × serum albumin [g/L]) + (41.7 × [ABW (kg)/IBW (kg)]). IBW was determined as follows: for males, H (height) (cm) – 100 – [(H (cm) – 150)/4]; for females, H (cm) – 100 – [(H (cm) – 150)/2.5]. In cases where the actual weight exceeded the ideal weight, the ratio of actual to ideal weight was set to 1. Consistent with previous studies (14–16), participants were divided into two groups: GNRI-defined nutritional risk group (GNRI ≤98) and non-nutritional risk group (GNRI >98).

Table 1 lists the full computational formulas for all 12 CBC-derived hematological inflammatory ratios analyzed in this study for transparent variable definition. These 12 hematological indices were all derived from baseline admission CBC results, and we acknowledge that multiple ratios share identical raw blood cell parameters (lymphocytes, monocytes, neutrophils, platelets, hemoglobin, albumin), creating inherent mathematical correlation. Since GNRI incorporates serum albumin and body weight metrics, the associations between these CBC-derived ratios and GNRI-defined nutritional risk may be mediated indirectly through shared albumin-related metabolic signals, systemic inflammatory activation linked to nutritional depletion, or body composition differences, rather than representing fully independent nutritional biomarkers unconfounded by albumin and body composition. We addressed this confounding pathway in subsequent stratified regression and multicollinearity diagnostics to distinguish direct and indirect correlations between each ratio and nutritional risk. Since GNRI relies on albumin and anthropometric data, these hematologic ratios may correlate with GNRI indirectly via inflammation, body composition or albumin metabolism rather than independent nutritional biomarkers.

**Table 1:** Formula of 12 CBC-derived hematological inflammatory ratios.

Abbreviation	Full name	Calculation formula
LR	Lymphocyte ratio	Lymphocyte count/total white blood cell count
RLR	Red blood cell-to-lymphocyte ratio	Red blood cell count/lymphocyte count
HLR	Hemoglobin-to-lymphocyte ratio	Hemoglobin concentration/lymphocyte count
MLR	Monocyte-to-lymphocyte ratio	Monocyte count/lymphocyte count
MNR	Monocyte-to-neutrophil ratio	Monocyte count/neutrophil count
NLR	Neutrophil-to-lymphocyte ratio	Neutrophil count/lymphocyte count
PLR	Platelet-to-lymphocyte ratio	Platelet count/lymphocyte count
PNR	Platelet-to-neutrophil ratio	Platelet count/neutrophil count
NLR/PLT*100	NLR-to-platelet ratio (×100)	(Neutrophil count/lymphocyte count)/platelet count × 100
MLR/PLT	MLR-to-platelet ratio	(Monocyte count/lymphocyte count)/platelet count
MPVLR	Mean platelet volume-to-lymphocyte ratio	Mean platelet volume/lymphocyte count
ALBLR	Serum albumin-to-lymphocyte ratio	Serum albumin concentration/lymphocyte count

### 2.3 Statistical analysis

Statistical analyses were performed using SPSS version 27.0 (IBM Corp., Armonk, NY, United States) and R version 4.0.2 (R Foundation for Statistical Computing, Vienna, Austria). Continuous variables were presented as mean ± standard deviation (SD) if normally distributed and compared using the independent samples t-test; non-normal continuous data were reported as median (interquartile range, IQR) and compared with the Mann–Whitney *U* test. Categorical variables were summarized as counts and percentages, with intergroup comparisons conducted via the Chi-square test. Baseline clinical and hematological indicators were first compared between the GNRI-defined nutritional risk group and non-risk group to identify univariate candidate correlates for subsequent regression modeling. Three distinct regression frameworks were applied with clear separate rationales to avoid interpretive confusion across overlapping models: ① forward stepwise binary logistic regression (primary unadjusted screening model): This model was only used for preliminary univariate screening of potential associated factors by incorporating all statistically significant univariate variables; given the high risk of multicollinearity among mathematically correlated hematological ratios, we supplemented variance inflation factor (VIF) diagnostics for all variables entered into the model to quantify collinearity severity. Any indicator with VIF > 10 was excluded from the final stepwise output to mitigate unstable odds ratio (OR) estimates driven by shared blood cell denominators/numerators across ratios. ② Three hierarchical fully adjusted logistic regression models: These sequential models were constructed to gradually adjust for distinct sets of clinical confounders, with the core goal of verifying whether CBC-derived hematological inflammatory ratios retained independent associations after progressive correction of demographic, hemodynamic, metabolic, and inflammatory covariates. Each hierarchical model also reported corresponding VIF values for all included hematological markers to monitor multicollinearity. ③ Incremental correlative performance comparison models: Base clinical model (age, hypertension history, ALT, UA, TG) was compared against extended models adding single or combined CBC-derived hematological inflammatory ratios, using C-statistic (AUC), DeLong’s test, likelihood ratio test and Akaike Information Criterion (AIC) to quantify incremental discriminative value contributed by hematological ratios beyond standard clinical covariates alone.

Multicollinearity diagnosis was a mandatory supplementary step for all multivariate regression analyses in this study. Due to overlapping raw blood cell counts across the 12 CBC-derived hematological inflammatory ratios (lymphocytes, monocytes, neutrophils, platelets shared across multiple indices), high inter-variable correlation and collinearity risk existed. VIF values were calculated for every predictor included in each regression framework; variables with VIF exceeding 10 were eliminated to reduce biased, unstable OR values and overly wide 95% confidence intervals (CIs). For this reason, highly correlated paired ratios were not simultaneously retained within the same regression equation where collinearity thresholds were breached. Receiver operating characteristic (ROC) curve analysis was performed to evaluate the discriminative ability of the combined clinical-biomarker model for GNRI-defined nutritional risk. The Hosmer–Lemeshow goodness-of-fit test was adopted to assess calibration of all binary logistic regression models. No internal validation (bootstrap resampling, cross-validation, or training/validation dataset split) was performed in the current single-center retrospective cohort, which represents an acknowledged major limitation of this exploratory risk stratification model; all correlative results are interpreted cautiously without claims of stable generalizability to external cohorts. Consistent with statistical caution suggested by wide CIs for MLR-derived ORs across regression models, all results avoid absolute descriptive language such as “most robust predictor.” Instead, the standardized neutral phrasing “MLR showed the strongest association within this study’s dataset” is used throughout Results and Discussion sections to reflect limited within-sample evidence without overgeneralization of correlative superiority. Statistical significance threshold was set at two-tailed *p* < 0.05 for all analyses.

### 2.4 Ethical approval

The study was approved by the Ethics Committee of Ruian People’s Hospital (Approval No.: [YJ2024038]), and all research procedures complied with the Declaration of Helsinki. Written informed consent was obtained from all participants or their legal surrogates prior to data extraction.

## 3 Results

### 3.1 Baseline characteristics of the study subjects

A total of 1,097 patients with acute ischemic stroke (AIS) were screened, and 540 elderly patients were finally included in the analysis after applying exclusion criteria (Figure 1). Of the total cohort, 245 patients (45.4%) met the GNRI ≤98 threshold and were classified as having GNRI-defined nutritional risk, while the remaining 295 patients (54.6%) were categorized as non-nutritional risk patients. Significant differences were observed between the two groups in terms of demographic and clinical parameters. Patients in the malnutrition risk group were significantly older than those in the non-nutritional risk group (median age: 78.00 [IQR: 70.50–83.50] vs. 72.00 [IQR: 67.00–80.00] years, *p* < 0.001). Regarding comorbidities, the prevalence of hypertension was significantly lower in the malnutrition risk group compared to the non-nutritional risk group (77.1% vs. 86.1%, *p* = 0.007). Physiologically, patients at risk of malnutrition presented with lower diastolic blood pressure (DBP) (median: 79.00 vs. 82.00 mmHg, *p* = 0.005). Furthermore, several metabolic and biochemical markers were significantly lower in the malnutrition risk group, including alanine aminotransferase (ALT) (14.00 vs. 18.00 U/L, *p* < 0.001), uric acid (UA) (295.00 vs. 319.00 μmol/L, *p* = 0.002), and triglycerides (TG) (1.04 vs. 1.29 mmol/L, *p* < 0.001). There were no statistically significant differences between the two groups regarding gender, other comorbidities (hyperlipidemia, diabetes, atrial fibrillation), systolic blood pressure (SBP), length of stay (LOS), or other lipid profile parameters (TC, HDL, LDL) (all *p* > 0.05). Several key stroke-related clinical parameters, including National Institutes of Health Stroke Scale (NIHSS) score, infarct volume, infarct location, dysphagia/aspiration risk, pre-stroke functional status, reperfusion therapy status, and feeding status at admission, were not consistently documented across the complete electronic medical records of all enrolled patients, and therefore were not available for subgroup stratification and multivariable adjustment (Table 2).

**Table 2:** Baseline characteristics of elderly AIS patients stratified by GNRI.

Variables	Malnutrition risk (*n* = 245)	Non-nutritional risk (*n* = 295)	*p*-value
Age (y)	78.00(70.50 ~ 83.50)	72.00(67.00 ~ 80.00)	<0.001
Male (%)	133(54.3)	169(57.3)	0.484
Hypertension	189(77.1)	254(86.1)	0.007
Hyperlipidemia	24(9.8)	44(14.9)	0.074
Diabetes	96(39.2)	131(44.4)	0.221
AF	41(16.7)	43(14.6)	0.491
SBP (mmHg)	156.00(142.00 ~ 175.00)	160.00(145.00 ~ 176.00)	0.343
DBP (mmHg)	79.00(70.00 ~ 87.00)	82.00(74.00 ~ 90.00)	0.005
LOS (d)	12.00(8.00 ~ 16.00)	12.00(9.00 ~ 16.00)	1.000
ALT (U/L)	14.00(10.00 ~ 19.00)	18.00(13.00 ~ 25.00)	<0.001
AST (U/L)	20.00(16.00 ~ 24.50)	21.00(17.00 ~ 27.00)	0.086
Cr (μmol/L)	69.00(61.00 ~ 83.50)	69.00(61.00 ~ 80.00)	0.667
UA (μmol/L)	295.00(231.50 ~ 361.50)	319.00(260.00 ~ 379.00)	0.002
TC (mmol/L)	4.01(3.37 ~ 4.62)	4.19(3.42 ~ 5.00)	0.104
TG (mmol/L)	1.04(0.81 ~ 1.48)	1.29(1.00 ~ 1.73)	<0.001
HDL (mmol/L)	0.98(0.84 ~ 1.14)	0.98(0.83 ~ 1.16)	0.797
LDL (mmol/L)	2.40(1.89 ~ 3.04)	2.49(1.80 ~ 3.19)	0.591

Data are presented as median (interquartile range) for continuous variables and *n* (%) for categorical variables. *p* < 0.05 was considered statistically significant. AIS, acute ischemic stroke; GNRI, Geriatric Nutritional Risk Index; ALT, alanine aminotransferase; UA, uric acid; TG, triglyceride; NLR, neutrophil-to-lymphocyte ratio; MLR, monocyte-to-lymphocyte ratio; PLR, platelet-to-lymphocyte ratio; SBP, systolic blood pressure; DBP, diastolic blood pressure.

### 3.2 Comparison of lymphocyte-related ratios between groups

We additionally compared raw uncombined levels of lymphocytes, monocytes, neutrophils, platelets, hemoglobin and albumin between groups to separate effects of individual cell counts from composite ratios. The LR was significantly lower in the malnutrition risk group compared to the non-nutritional risk group (0.23 ± 0.093 vs. 0.26 ± 0.088, *p* < 0.001), reflecting a state of relative lymphopenia associated with poor nutritional status. Conversely, most composite inflammatory ratios were significantly elevated in the malnutrition risk group. Specifically, the MLR (median: 0.38 vs. 0.32, *p* < 0.001), RLR (2.84 vs. 2.67, *p* = 0.045), NLR (3.00 vs. 2.50, *p* < 0.001), PLR (143.08 vs. 121.67, *p* < 0.001) and MPVLR (7.93 vs. 6.93, *p* < 0.001) were all significantly higher in patients with GNRI≤98. Furthermore, indicators adjusted for platelet count, including NLR/PLT*100 (1.429 vs. 1.252, *p* = 0.002) and MLR/PLT (0.19 vs. 0.15, *p* < 0.001), were significantly elevated in the malnutrition risk group. No statistically significant differences were found between the two groups regarding HLR, MNR, PNR, or ALBLR (all *p* > 0.05). However, 12 separate hematologic ratio variables were compared in univariate intergroup analyses, substantially increasing the risk of Type I error due to multiple comparisons. All univariate between-group comparisons should be interpreted as exploratory preliminary associations rather than definitive independent differences; formal multivariate modeling was employed to mitigate multiple comparison bias and filter out spurious univariate correlations attributable to random testing fluctuations (Table 3).

**Table 3:** Comparison of inflammatory hematological indicators in elderly AIS patients by GNRI category.

Variables	Malnutrition risk (*n* = 245)	Non-nutritional risk (*n* = 295)	*P*-value
LR	0.23 ± 0.093	0.26 ± 0.088	<0.001
HLR	87.69(66.56 ~ 113.15)	82.78(66.00 ~ 105.71)	0.100
RLR	2.84(2.21 ~ 3.77)	2.67(2.18 ~ 3.32)	0.045
MLR	0.38(0.29 ~ 0.54)	0.32(0.24 ~ 0.42)	<0.001
NLR	3.00(1.98 ~ 4.91)	2.50(1.81 ~ 3.65)	<0.001
MNR	0.13(0.09 ~ 0.17)	0.12(0.10 ~ 0.15)	0.510
NLR/PLT*100	1.429(1.029 ~ 2.474)	1.252(0.861 ~ 1.901)	0.002
MLR/PLT	0.19(0.13 ~ 0.30)	0.15(0.11 ~ 0.22)	<0.001
PNR	48.73(33.56 ~ 64.71)	50.24(36.67 ~ 63.44)	0.350
ALBLR	25.00(19.75 ~ 34.15)	24.40(19.60 ~ 30.25)	0.139
PLR	143.08(109.46 ~ 197.74)	121.67(96.47 ~ 162.86)	<0.001
MPVLR	7.93(5.86 ~ 11.05)	6.93(5.38 ~ 8.85)	<0.001

Data are presented as mean ± standard deviation for continuous variables with normal distribution (LR) and median (interquartile range) for continuous variables with non-normal distribution (HLR, RLR, MLR, NLR, MNR, NLR/PLT*100, MLR/PLT, PNR, ALBLR, PLR, MPVLR). Comparisons between the malnutrition risk group and non-nutritional risk group were performed using the independent samples t-test (for normally distributed variables) and Mann–Whitney U test (for non-normally distributed variables). *p* < 0.05 was considered statistically significant. GNRI, Geriatric Nutritional Risk Index; AIS, acute ischemic stroke; LR, lymphocyte ratio; HLR, hemoglobin-to-lymphocyte ratio; RLR, red blood cell-to-lymphocyte ratio; MLR, monocyte-to-lymphocyte ratio; NLR, neutrophil-to-lymphocyte ratio; MNR, monocyte-to-neutrophil ratio; PLT, platelet count; PNR, platelet-to-neutrophil ratio; ALBLR, albumin-to-lymphocyte ratio; PLR, platelet-to-lymphocyte ratio; MPVLR, mean platelet volume-to-lymphocyte ratio.

### 3.3 Binary logistic regression analysis of independent correlates for nutritional risk

Using the forward stepwise (Likelihood Ratio) method, a multivariate binary logistic regression model was constructed with nutritional risk status as the dependent variable. Covariates entered into the model included age, hypertension history, DBP, ALT, UA, TG, LR, RLR, MLR, NLR, NLR/PLT*100, MLR/PLT, PLR, and MPVLR. The results reveal several significant independent factors associated with nutritional status. The MLR emerged as the strongest independent risk factor for malnutrition.

Multivariate regression demonstrated MLR was the correlate with the strongest point estimate of association within this single cohort dataset (Odds Ratio [OR] = 16.703, 95% CI: 4.303–64.834, *p* < 0.001). Notably, this association was only adjusted for all available demographic, hemodynamic, metabolic and routine hematological covariates; core stroke-specific confounders including NIHSS score, infarct volume, dysphagia, reperfusion therapy and pre-stroke functional status were missing in electronic medical records and could not be incorporated into regression adjustment, so substantial residual confounding cannot be fully eliminated in the current analysis. Additionally, advanced Age was confirmed as an independent risk factor, with the risk increasing by 4.1% for each additional year of age (OR = 1.041, 95% CI: 1.016–1.067, *p* = 0.001). A history of Hypertension was also independently associated with a higher risk of malnutrition (OR = 1.690, 95% CI: 1.029–2.774, *p* = 0.038). Conversely, several metabolic indicators were identified as independent protective factors. Higher levels of ALT (OR = 0.965, 95% CI: 0.946–0.983, *p* < 0.001), Uric Acid (UA) (OR = 0.997, 95% CI: 0.995–0.999, *p* = 0.003), and Triglycerides (TG) (OR = 0.670, 95% CI: 0.493–0.910, *p* = 0.010) were all significantly associated with a reduced risk of malnutrition. The Hosmer–Lemeshow test confirmed adequate model calibration (χ^2^ = 10.227, df = 8, *p* = 0.249). Interestingly, the novel index NLR/PLT*100 also showed a protective association in the multivariate model (OR = 0.876, 95% CI: 0.782–0.982, *p* = 0.023), contrasting with its univariate elevation, which suggests a complex interaction with other platelet-related variables. This directional reversal may be partially attributable to multicollinearity between NLR/PLT × 100 and other shared cell-count ratios within the model, as well as statistical suppression effects arising from co-adjusted inflammatory markers, rather than reflecting a consistent biological protective effect (Table 4).

**Table 4:** Binary logistic regression analysis in patients with malnutrition or nutritional risk.

Variables	OR	*P*-value	95%CI
MLR	16.703	<0.001	(4.303, 64.834)
NLR/PLT*100	0.876	0.023	(0.782, 0.982)
Age	1.041	0.001	(1.016, 1.067)
Hypertension	1.690	0.038	(1.029, 2.774)
ALT	0.965	<0.001	(0.946, 0.983)
UA	0.997	0.003	(0.995, 0.999)
TG	0.670	0.010	(0.493, 0.910)

Data are presented as odds ratio (OR) with 95% confidence interval (95%CI) and corresponding p values. Binary logistic regression analysis was performed to identify independent correlates of malnutrition or nutritional risk in elderly patients with acute ischemic stroke (AIS). *p* < 0.05 was considered statistically significant. MLR, monocyte-to-lymphocyte ratio; NLR, neutrophil-to-lymphocyte ratio; PLT, platelet count; ALT, alanine aminotransferase; UA, uric acid; TG, triglyceride; OR, odds ratio; CI, confidence interval.

### 3.4 Construction and within-sample performance evaluation of the exploratory nutritional risk model

A prediction model for nutritional risk in elderly patients with AIS was established by incorporating age, history of hypertension, ALT, UA, TG, MLR, and NLR/PLT*100. ROC curve analysis demonstrated that the model achieved a sensitivity of 72.2% and specificity of 67.8% for nutritional risk screening, with an optimal cut-off value of 0.435 determined by the maximum Youden index. The area under the ROC curve (AUC) was 0.735 (95% CI: 0.693–0.778), indicating moderate discriminative ability. Collectively, the comprehensive model exhibited relatively optimal correlative performance under the current study conditions (Figure 2). This single-center retrospective study did not employ internal bootstrap resampling, cross-validation or external independent cohort validation; all AUCs, sensitivities, specificities, and optimal cutoff values derived from ROC curves reflect only the apparent discriminative performance within the original modeling sample and cannot represent the generalizability of the predictive validity to external populations.

**Figure 2:**
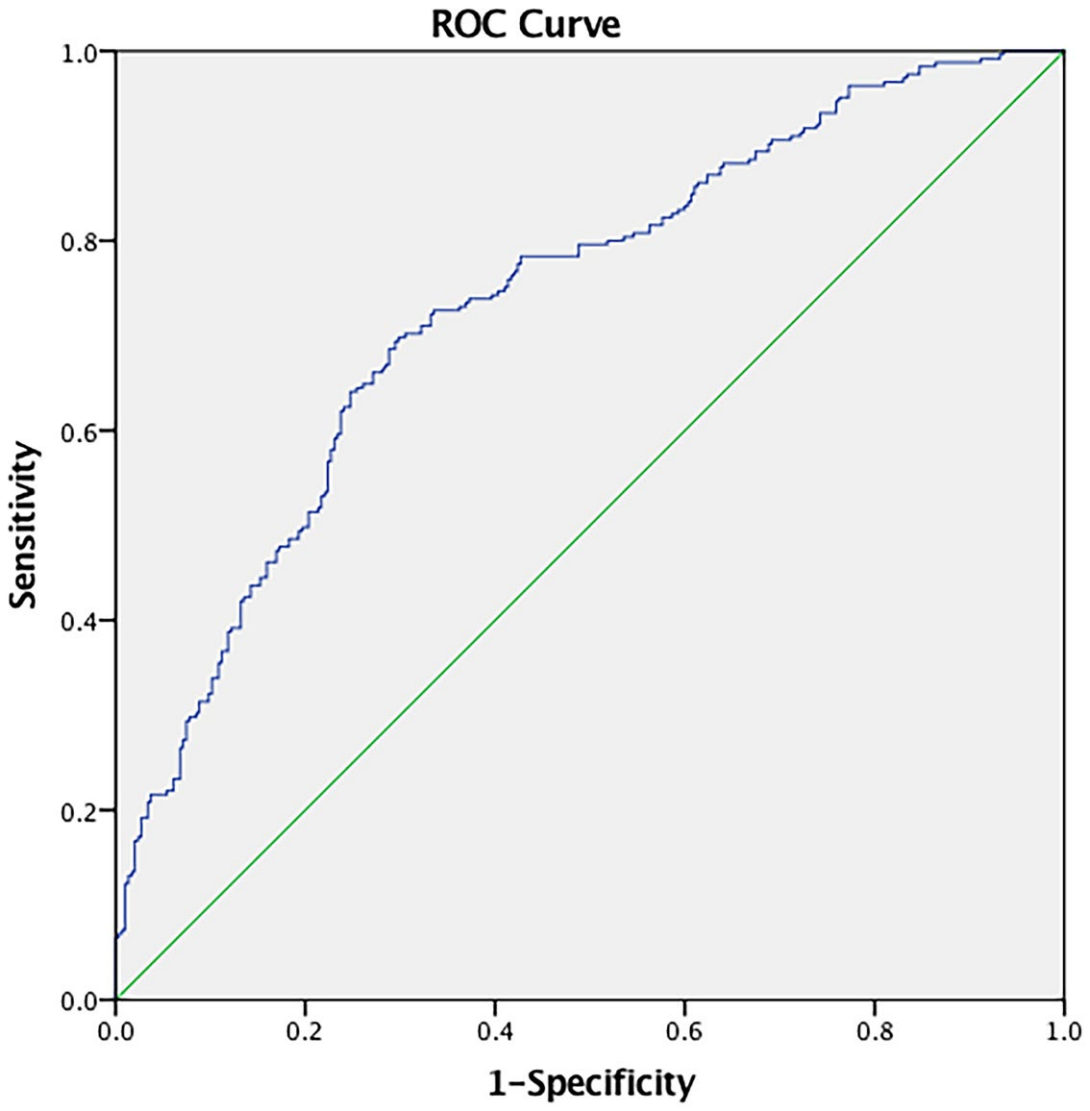
ROC curve analysis of the nutritional risk prediction model in elderly patients with acute ischemic stroke (AIS). The curve represents the composite model integrating the Base Model [age, hypertension, alanine aminotransferase (ALT), uric acid (UA), triglyceride (TG)], monocyte-to-lymphocyte ratio (MLR), and NLR/PLT*100 (neutrophil-to-lymphocyte ratio divided by platelet count and multiplied by 100). The area under the curve (AUC) was 0.735 with a 95% confidence interval (CI) of 0.693–0.778; the optimal cutoff value of the model was 0.435, with a sensitivity of 72.2% and a specificity of 67.8%.

### 3.5 Adjusted logistic regression models for factors associated with nutritional risk in elderly AIS patients

The results of the adjusted logistic regression analyses for factors associated with nutritional risk in elderly AIS patients, using three hierarchical models to control for potential confounders. Across all three models, MLR, RLR, and MPVLR consistently demonstrated significant independent associations with nutritional risk. Notably, MLR exhibited an extremely strong positive association, with the highest Odds Ratios (ORs) observed in all models (Model 1: OR = 100.95, *p* = 0.012; Model 2: OR = 116.74, *p* = 0.009; Model 3: OR = 67.33, *p* = 0.015), highlighting its robust correlative value regardless of covariate adjustment. Again, all adjustments failed to include critical stroke-specific confounders missing from medical records, which may introduce unmeasured residual confounding and bias effect size estimation. Similarly, MPVLR remained a significant risk factor across all models (ORs ranging from 1.305 to 1.370, all *p* < 0.001). Interestingly, RLR showed a significant negative association (protective effect) in the multivariate models (ORs ranging from 0.382 to 0.414, all *p* < 0.001), this opposing direction represents a statistical suppression effect driven by co-adjusted overlapping inflammatory ratio variables and multicollinearity between shared blood cell denominators and numerators.

Notably, NLR/PLT*100 lost statistical significance (all *p* > 0.05) in all three fully adjusted hierarchical models after comprehensive covariate control, reframing our interpretation that this index does not retain consistent independent association after broad correction for inflammatory and metabolic confounders. The Hosmer–Lemeshow test confirmed acceptable calibration for each hierarchical model (all *p* > 0.05) (Table 5).

**Table 5:** Adjusted logistic regression models for factors associated with nutritional risk in elderly AIS patients.

Variables	Model 1		Model 2		Model 3	
	OR (95%CI)	*p*-value	OR (95%CI)	*P*-value	OR (95%CI)	*P*-value
Age	1.035(1.009–1.061)	0.008	1.036(1.011–1.063)	0.005		
DBP	0.994(0.979–1.010)	0.441				
Hypertension	1.818(1.087–3.043)	0.023	1.846(1.106–3.082)	0.019		
ALT	0.967(0.949–0.986)	0.001	0.967(0.949–0.985)	<0.001		
UA	0.997(0.995–0.999)	0.002	0.997(0.995–0.999)	0.002		
TG	0.699(0.509–0.959)	0.027	0.702(0.512–0.963)	0.028		
LR	0.743(0.028–19.350)	0.858	0.827(0.032–21.306)	0.909	0.404(0.017–9.332)	0.572
RLR	0.414(0.268–0.637)	<0.001	0.400(0.262–0.612)	<0.001	0.382(0.257–0.569)	<0.001
MLR	100.95(2.747–3710.399)	0.012	116.742(3.232–4216.532)	0.009	67.326(2.280–1987.752)	0.015
NLR	0.801(0.589–1.090)	0.159	0.794(0.584–1.080)	0.142	0.806(0.599–1.085)	0.156
NLR/PLT*100	1.274(0.720–2.256)	0.406	1.297(0.720–2.256)	0.368	1.201(0.698–2.066)	0.508
MLR/PLT	0.058(0.000–22.468)	0.348	0.046(<0.001–17.051)	0.307	0.133(0.000–38.431)	0.485
PLR	1.004(0.999–1.008)	0.112	1.004(0.999–1.008)	0.108	1.004(1.000–1.008)	0.062
MPVLR	1.305(1.129–1.508)	<0.001	1.318(1.142–1.520)	<0.001	1.370(1.200–1.565)	<0.001

Data are presented as odds ratio (OR) with 95% confidence interval (95%CI) and corresponding *p*-values. Three adjusted logistic regression models were constructed to analyze the association between relevant factors and nutritional risk in elderly patients with acute ischemic stroke (AIS): Model 1 included age, diastolic blood pressure (DBP), hypertension, alanine aminotransferase (ALT), uric acid (UA), triglyceride (TG), and all inflammatory hematological indicators; Model 2 excluded DBP based on Model 1; Model 3 only included lymphocyte ratio (LR), red blood cell-to-lymphocyte ratio (RLR), monocyte-to-lymphocyte ratio (MLR), neutrophil-to-lymphocyte ratio (NLR), NLR/PLT*100, MLR/PLT, platelet-to-lymphocyte ratio (PLR), and mean platelet volume-to-lymphocyte ratio (MPVLR). p < 0.05 was considered statistically significant. OR, odds ratio; CI, confidence interval; AIS, acute ischemic stroke; DBP, diastolic blood pressure; ALT, alanine aminotransferase; UA, uric acid; TG, triglyceride; LR, lymphocyte ratio; RLR, red blood cell-to-lymphocyte ratio; MLR, monocyte-to-lymphocyte ratio; NLR, neutrophil-to-lymphocyte ratio; PLT, platelet count; PLR, platelet-to-lymphocyte ratio; MPVLR, mean platelet volume-to-lymphocyte ratio.

### 3.6 Some lymphocyte-related ratios improve the models

To evaluate the incremental correlative value of CBC-derived hematological inflammatory ratios over traditional clinical risk factors, we compared the Base Model (comprising age, hypertension, ALT, UA, and TG) with models incorporating additional lymphocyte-related ratios. The model performance was assessed using the C-statistic, likelihood ratio test and Akaike Information Criterion (AIC). The Base Model yielded a C-statistic of 0.710 (95% CI: 0.667–0.753). Among the single-indicator models, the addition of MLR significantly improved the model’s discriminatory power, increasing the C-statistic to 0.730 (95% CI: 0.688–0.773). This improvement was statistically significant (DeLong’s test *p* = 0.020). Furthermore, the likelihood ratio test confirmed that the model including MLR fit the data significantly better than the Base Model (LLR improvement = 7.302, *p* = 0.007), and it resulted in a lower AIC (662.376 vs. 675.702), indicating a better goodness-of-fit.

In contrast, the inclusion of other individual ratios (LR, RLR, NLR, NLR/PLT*100, MLR/PLT, PLR, or MPVLR) did not lead to statistically significant improvements in the C-statistic compared to the Base Model (all DeLong’s test *p* > 0.05), nor did they show significance in the likelihood ratio test. Notably, the combined model incorporating both MLR and NLR/PLT*100 achieved the highest correlative performance, with a C-statistic of 0.735 (95% CI: 0.693–0.778, DeLong’s test *p* = 0.010). This model also demonstrated the most substantial LLR improvement (8.022, *p* = 0.018) and the lowest AIC value (659.082), suggesting it is the optimal model for identifying nutritional risk in this cohort (Table 6).

**Table 6:** Incremental correlative value of CBC-derived hematological inflammatory ratios.

Models	*C* statistic			LR test			AIC
	Estimate (95%CI)	*p*-value (ROC)	P value (DeLong’s test)	LLR	LLR improvement from base model	*P*-value	
Base model	0.710(0.667–0.753)	<0.001	Reference	57.509	Reference	Reference	675.702
Base model + Lr	0.718(0.675–0.761)	<0.001	0.200	59.319	1.810	0.179	672.326
Base model + RLR	0.710(0.667–0.753)	<0.001	0.808	58.622	1.113	0.291	677.294
Base model + MLR	0.730(0.688–0.773)	<0.001	0.020	64.811	7.302	0.007	662.376
Base model + NLR	0.714(0.671–0.757)	<0.001	0.238	58.466	0.957	0.328	676.014
Base model + NLR/PLT*100	0.711(0.668–0.754)	<0.001	0.638	58.724	1.215	0.270	676.788
Base model + MLR/PLT	0.719(0.676–0.762)	<0.001	0.093	59.384	1.875	0.171	672.670
Base model + PLR	0.720(0.677–0.762)	<0.001	0.076	59.868	2.359	0.125	672.591
Base model + MPVLR	0.718(0.676–0.761)	<0.001	0.082	59.659	2.150	0.143	673.916
Base model + MLR + NLR/PLT*100	0.735(0.693–0.778)	<0.001	0.010	65.531	8.022	0.018	659.082

Data are presented as C statistic (with 95% confidence interval [95%CI]), likelihood ratio (LLR), LLR improvement from the base model, and Akaike information criterion (AIC) values. This table was used to evaluate the incremental correlative value of CBC-derived hematological inflammatory ratios for nutritional risk in elderly patients with acute ischemic stroke (AIS). The base model included age, hypertension, alanine aminotransferase (ALT), uric acid (UA), and triglyceride (TG). The C statistic was used to assess the correlative efficacy of each model, with DeLong’s test used to compare differences in C statistics between models; the likelihood ratio (LR) test was used to evaluate the statistical significance of model improvement; AIC was used to assess model fitting degree (lower AIC values indicate better model fitting). *p* < 0.05 was considered statistically significant. AIS, acute ischemic stroke; CI, confidence interval; LLR, likelihood ratio; AIC, Akaike information criterion; ALT, alanine aminotransferase; UA, uric acid; TG, triglyceride; LR, lymphocyte ratio; RLR, red blood cell-to-lymphocyte ratio; MLR, monocyte-to-lymphocyte ratio; NLR, neutrophil-to-lymphocyte ratio; PLT, platelet count; PLR, platelet-to-lymphocyte ratio; MPVLR, mean platelet volume-to-lymphocyte ratio.

## 4 Discussion

To the best of our knowledge, this is the first study to comprehensively analyze cross-sectional associations between a full panel of CBC-derived hematological inflammatory ratios and GNRI-defined nutritional risk among elderly patients with AIS. By evaluating 12 CBC-derived indices simultaneously, we sought to identify markers with strong within-sample correlations and build an exploratory multivariate model for risk stratification reference. Our correlative analyses demonstrate that MLR exhibits the strongest within-cohort association with GNRI-defined nutritional risk among all tested ratios. Furthermore, the multivariate model combining baseline clinical covariates, MLR and NLR/PLT*100 showed modest incremental discrimination compared to the clinical base model only within this single cohort. These findings fill existing literature gaps regarding the correlation spectrum of lymphocyte-related ratios and admission nutritional risk in elderly stroke patients, yet the model’s clinical applicability requires extremely cautious interpretation given multiple methodological limitations.

Malnutrition is primarily characterized by alterations in body composition and a depletion of body cell mass resulting from inadequate nutrient intake (17). As a critical predictor of morbidity and mortality, malnutrition may precipitate impairments in muscle and immune function, and contribute to reduced bone mass, anemia, cognitive dysfunction, and impaired wound healing (18). Malnutrition can directly lead to a decrease in the count of peripheral blood lymphocytes by inhibiting the proliferation and differentiation of lymphocytes in the bone marrow and accelerating their apoptosis; and the reduction of lymphocytes will further weaken the immune defense function of the body, forming a vicious cycle where malnutrition and immunosuppression intensify each other. In the context of AIS, post-stroke malnutrition has been demonstrated to significantly increase the risk of AIS-related complications, such as pulmonary and urinary tract infections, deep vein thrombosis, and gastrointestinal bleeding, thereby prolonging hospitalization and adversely affecting recovery and long-term prognosis. Therefore, early and accurate nutritional assessment is essential for elderly AIS patients (19). Notably, the clinical manifestations of malnutrition are often subtle and easily overlooked. Consequently, at-risk subjects should be identified using validated screening tools to facilitate timely and appropriate assessment and intervention (15, 20). In this regard, the GNRI serves as a robust nutritional status indicator. By integrating objective parameters such as body weight, height, and serum albumin levels, the GNRI provides a simple, well-established, and accurate nutritional screening instrument that is particularly suitable for elderly populations. In the present study, participants were categorized into two groups based on their GNRI scores. Our findings revealed that 45.4% of elderly AIS patients were identified as being at nutritional risk, a figure that aligns with the high prevalence of malnutrition previously reported in stroke literature (21). This high prevalence underscores the imperative for targeted nutritional screening within this specific population (22). Analysis of baseline characteristics indicated that patients at nutritional risk were of more advanced age, exhibited a lower prevalence of hypertension, and presented with significantly reduced levels of ALT, UA, and TG. These results suggest that advanced age and altered metabolic profiles may serve as critical clinical indicators of nutritional risk. Such observations are consistent with the physiological decline associated with aging which predisposes individuals to nutritional imbalances, a vulnerability that becomes particularly pronounced following an acute cerebrovascular event (23).

The MLR, which is readily derived from routine blood parameters, has emerged as a promising inflammatory marker (24, 25). Specifically, MLR has been demonstrated to reflect disease activity in patients with ulcerative colitis (26). In the AIS population, several investigations have confirmed its prognostic value; for instance, Zhu et al. (27) identified its association with unfavorable clinical outcomes. Similarly, Ding et al. (28) demonstrated that MLR serves as an independent risk factor for post-stroke depression. Nevertheless, evidence regarding the association between MLR and nutritional risk in elderly AIS patients remains sparse. Therefore, to the best of our knowledge, this is the first investigation to investigate the relationship between MLR and nutritional status, as assessed by the GNRI, in this specific population.

Our findings revealed that MLR levels were significantly elevated in patients with nutritional risk compared to those without risk. Furthermore, Spearman correlation analysis indicated a significant inverse correlation between MLR and GNRI scores. Subsequent multivariate logistic regression analysis confirmed MLR as an independent predictor of nutritional risk in elderly after adjustment for all available clinical covariates, with residual confounding from unrecorded stroke severity, dysphagia and reperfusion therapy uneliminated, which integrates monocyte-mediated systemic inflammation with lymphocyte-related immune function. Monocytes are fundamental components of the innate immune system and serve as key drivers of the inflammatory response, which is often exacerbated in the context of AIS and aging (a phenomenon termed ‘inflammaging’) (29). Conversely, the lymphocyte count is a well-established marker of nutritional reserve and adaptive immune competence. Malnutrition compromises the immune system, leading to lymphopenia through mechanisms such as impaired lymphopoiesis and increased apoptosis (30). Consequently, an elevated MLR effectively captures the ‘dual burden’ of heightened pro-inflammatory activity and impaired nutritional/immune reserves. While previous studies have identified the MLR as a prognostic marker for stroke severity and malignancies, our results extend its clinical utility to the domain of nutritional assessment, highlighting its potential as an objective surrogate marker when a comprehensive nutritional screening is not immediately feasible (7, 31). Notably, the MLR is derived from routine complete blood count (CBC) tests, offering the advantages of rapid acquisition, low cost, and non-invasiveness, making it highly suitable for routine clinical application, even in resource-limited settings.

In the present study, univariate analysis revealed significantly reduced Lr in the malnutrition risk group compared to the non-nutritional risk group, a finding potentially attributable to malnutrition-induced impairment of lymphopoiesis and increased apoptosis. Conversely, the NLR, PLR, MPVLR, RLR, and MLR/PLT were significantly elevated, all of which exhibited significant negative correlations with GNRI scores. The observed elevation in NLR aligns with previous findings reported by Kaya et al. (32). We observed contradictory correlative trends for NLR/PLT100: higher values correlated with nutritional risk in univariate analysis, yet showed a negative correlative coefficient in the stepwise logistic model. This inconsistency is primarily attributed to multicollinearity bias shared across all lymphocyte composite ratios, alongside statistical suppression effects when multiple overlapping hematologic variables are co-adjusted in one regression equation, rather than representing independent biological protective effects (33). Our combined exploratory model integrating age, hypertension, ALT, UA, TG, MLR and NLR/PLT100 achieved an AUC of 0.735, which only corresponds to moderate discriminative capacity within this sample. This model cannot be recommended as a reliable standalone clinical screening tool for routine use, given several critical limitations, including unstable odds ratio estimates with extremely wide confidence intervals, absence of internal or external validation, and failure to adjust for core stroke-related confounders (e.g., NIHSS score, infarct volume, dysphagia, and reperfusion therapy). Although incorporating MLR and NLR/PLT*100 into the base model resulted in a statistically significant improvement in C-statistic (0.710 to 0.735), the absolute incremental gain in discriminative ability is modest, and its real-world clinical utility remains uncertain.

A notable novel finding of this study concerns the NLR-to-platelet ratio (NLR/PLT*100). While this index was significantly higher in the malnutrition risk group in univariate analysis, it emerged as an independent protective factor in multivariate regression. A nutritional risk prediction model for elderly patients with AIS was established by incorporating age, history of hypertension, ALT, UA, TG, MLR and NLR/PLT*100. The ROC curve analysis showed that the sensitivity of this model in screening nutritional risk was 72.2%, the specificity was 67.8%, and the optimal cut-off value determined based on the maximum Youden index was 0.435. The area under the ROC curve (AUC) was 0.735 (95% CI: 0.693–0.778). This difference indicates a non-linear relationship or complex interaction among neutrophil activation, platelet consumption and lymphocyte inhibition. It should be noted that the odds ratio (OR) value of ML in the multivariate corrected model of this study was relatively high and the 95% confidence interval was relatively wide, suggesting that the correlative effect of this indicator has a large variation and the model stability is limited. This phenomenon may be related to the sample size, weak collinearity among variables and the low event number to variable ratio (EPV), and is relatively common in small sample or multi-variable corrected models. Crucially, model comparison using the DeLong test and AIC demonstrated that incorporating NLR/PLT*100 into the MLR-enhanced model yielded the optimal correlative performance, characterized by the lowest AIC and highest log-likelihood ratio (LLR) improvement. This implies that NLR/PLT*100 provides complementary prognostic information, capturing residual risk unaccounted for by MLR alone and likely reflecting distinct pathophysiological pathways involving both coagulation and inflammation. The integration of MLR and NLR/PLT*100 significantly bolstered the correlative power of the base model (comprising age, hypertension, ALT, UA, and TG). These results suggest that integrating monocyte-lymphocyte balance with platelet-corrected neutrophil-lymphocyte balance more comprehensively captures the intricate crosstalk between inflammation, immunity, and nutritional status in elderly AIS patients, underscoring the value of multi-marker panels for risk stratification. This study highlights the clinical value of lymphocyte-related ratios (particularly MLR and NLR/PLT*100) as new independent correlates of nutritional risk in elderly AIS patients. Under the current research conditions, MLR has become the most robust individual indicator. After including MLR and NLR/PLT*100 in the baseline clinical model, the correlative performance has been optimized, with an AUC of 0.735, suggesting a moderate level of discrimination. Given its accessibility, cost-effectiveness, and non-invasive nature, these biomarkers can serve as an auxiliary screening tool for the early identification of nutritional risk in this vulnerable population. However, it should be noted that although the AUC of this model has statistical significance, an AUC value of 0.735 only represents a moderate level of discrimination. Meanwhile, NLR/PLT*100 was shown to be a protective factor for nutritional risk in the univariate stepwise regression model (OR = 0.876, *p* = 0.023), but did not reach statistical significance in the three multivariate adjusted models (all *p* > 0.05). This difference mainly results from the different numbers of covariates included in the stepwise regression model and the interactions between variables as well as potential weak collinearity in the multivariate adjusted models. The stepwise regression model only included some key variables and did not adequately control confounding factors; while the stratified model included more clinical and inflammatory indicators and corrected more confounding effects, resulting in a weakened independent effect of NLR/PLT*100 and a tendency toward non-significance. Additionally, this indicator has a certain correlation with other inflammatory ratios, and its correlative contribution in the multivariate model was covered by other stronger correlates (such as MLR). Therefore, in clinical application, this model should be interpreted with caution. It is more suitable as an auxiliary screening tool for nutritional risk rather than a decisive indicator for clinical comprehensive judgment.

However, it should be acknowledged that this study has several limitations. Firstly, this study is a single-center, cross-sectional observational study. The single-center design is prone to selection bias, which limits the generalizability and universality of the research conclusions. At the same time, the cross-sectional study design cannot establish the causal relationship between inflammation-related ratios and nutritional risk. Additionally, this study did not conduct internal validation (such as training set/validation set division, Bootstrap resampling, etc.), and the odds ratio (OR) of the multivariate adjustment model of MLR was too high (some models had OR > 100), suggesting possible insufficient model stability, multicollinearity, or overfitting risks. This study did not include key clinical confounding factors for correction, including stroke severity (NIHSS score), infarction volume, swallowing function status, and acute-phase specific treatment (intravenous thrombolysis, endovascular thrombectomy), etc. These factors are all core determinants affecting the nutritional status and clinical outcomes of patients with acute ischemic stroke. Their absence may lead to residual confounding and affect the clinical interpretability of the results. Therefore, future multi-center, prospective longitudinal studies should be conducted and internal and external validations should be carried out to further verify the findings of this study, clarify the underlying mechanisms, and promote the clinical translation and application of related biomarkers. At the same time, this study has a large sample size, which enables stratified analysis. To further verify the stability of the correlative efficacy of MLR, subsequent subgroup analyses can be conducted by stratifying by age (such as 60–75 years old, >75 years old), stroke severity (NIHSS score), and hypertension status, comparing the correlative consistency of MLR in different elderly subgroups, different stroke severities, and patients with or without hypertension, and exploring the interaction between inflammatory markers and stroke severity on nutritional risk. Moreover, the baseline of this study shows a difference in the prevalence of hypertension between the two groups. Stratified analysis can help further eliminate the confounding effect of hypertension and clarify the universality of the correlative value of ML.

## 5 Conclusion

This single-center retrospective cross-sectional study only identifies cross-sectional within-cohort associations between MLR, NLR/PLT100 and GNRI-defined nutritional risk among elderly AIS patients, rather than formally validated predictive markers for universal clinical application. Within this sample, MLR showed the strongest correlative estimate, while the combined clinical-biomarker model only delivered modest, moderate discrimination (AUC = 0.735) with limited incremental gain relative to base clinical variables. Due to critical methodological limitations, including unstable odds ratio estimates with wide confidence intervals, inherent multicollinearity among hematological indicators, insufficient adjustment for core stroke-related confounders, and absence of internal or external validation, this exploratory combined model is not yet adequate to serve as an effective tool for routine clinical risk stratification. Future large-scale, multicenter prospective cohort studies incorporating comprehensive stroke-specific clinical data, employing standardized multicollinearity control measures, and conducting formal model validation are warranted to confirm the reproducibility of these associations before clinical recommendations can be proposed.

## Data Availability

The original contributions presented in the study are included in the article/supplementary material, further inquiries can be directed to the corresponding author.

## Glossary

AIS: Acute ischemic stroke
LR: Lymphocyte ratio
RLR: Red blood cell-to-lymphocyte ratio
HLR: Hemoglobin-to-lymphocyte ratio
MLR: Monocyte-to-lymphocyte ratio
MNR: Monocyte-to-neutrophil ratio
NLR: Neutrophil-to-lymphocyte ratio
PLR: Platelet-to-lymphocyte ratio
PNR: Platelet-to-neutrophil ratio
NLR/PLT: NLR-to-platelet ratio
MLR/PLT: MLR-to-platelet ratio
MPVLR: Mean platelet volume-to-lymphocyte ratio
ALBLR: Albumin-to-lymphocyte ratio
GNRI: Geriatric Nutritional Risk Index
SBP: Systolic blood pressure
DBP: Diastolic blood pressure
ALT: Alanine aminotransferase
UA: Uric acid
TG: Triglyceride
TC: Total cholesterol
HDL: High-density lipoprotein
LDL: Low-density lipoprotein
OR: Odds ratio
CI: Confidence interval
IQR: Interquartile range
SD: Standard deviation
LOS: Length of stay
AF: Atrial fibrillation
CBC: Complete blood count
ROC: Receiver operating characteristic
AIC: Akaike Information Criterion
LLR: Log-likelihood ratio
